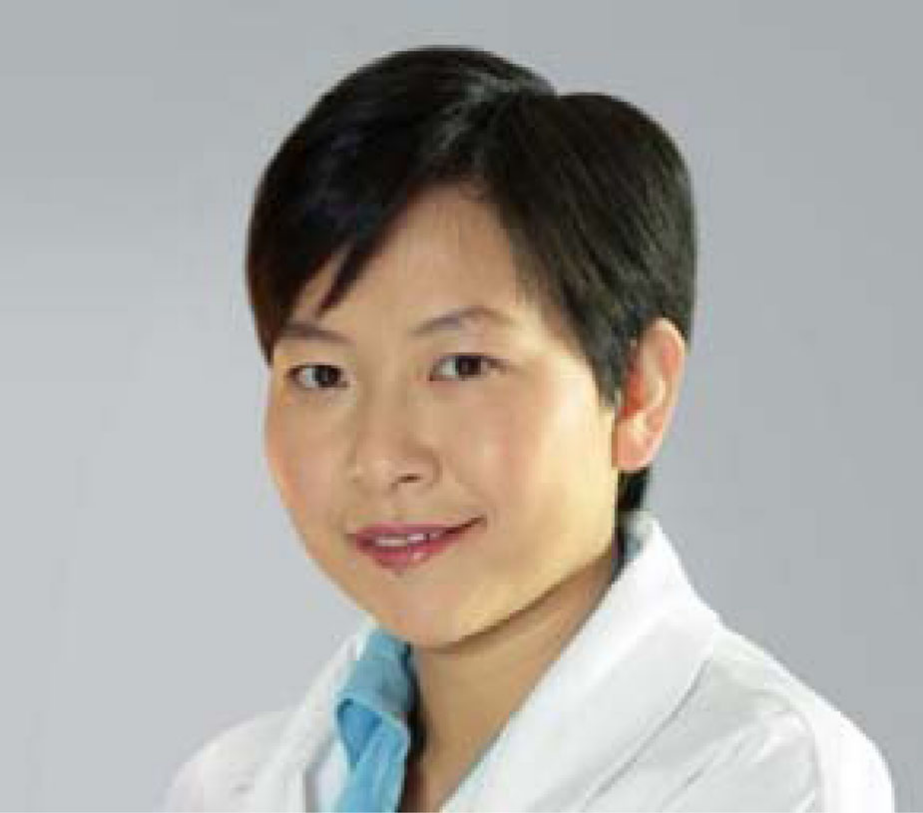# Light People: Professor Baohua Jia

**DOI:** 10.1038/s41377-022-00948-9

**Published:** 2022-08-31

**Authors:** Siqiu Guo

**Affiliations:** Light Publishing Group, Changchun Institute of Optics, Fine Mechanics and Physics, Chinese Academy of Sciences, 3888 Dong Nan Hu Road, Changchun, 130033 China

## Abstract

Integrated photonics means integrating multiple photonic functions on a single Photonic Integrated Chip (PIC). Empowered by various nanofabrication techniques on diverse innovative material platforms, remarkable advances have been made in integrated photonics in the last decade. *Light: Advanced Manufacturing (LAM)* is a new, highly selective, open-access, and free of charge international sister journal of the Nature Journal *Light: Science & Applications (Light)*. *LAM* aims to publish original innovative research papers and timely, state-of-the-art reviews in all modern areas of preferred light-based manufacturing, including fundamental and applied research as well as industrial innovations. *LAM* is organizing a special issue on integrated photonics, in order to capture the most exciting cutting edge advances in integrated photonics, including new material platforms, new fabrication and characterization technologies, new device architectures, new design principle of miniaturized components, nanophotonic devices, and their potential applications. We are very honored to feature Prof. Baohua Jia, the lead guest editor of this special issue, as this issue’s Light People. She is the Director of Center for Atomaterial Sciences and Technologies at Royal Melbourne Institute of Technology (RMIT) University and a top-level Future Fellow funded by the Australian Research Council. Her research focuses on fundamental light and nanomaterial interaction. In particular, her work on laser manipulation of two-dimensional materials has led to the design and fabrication of functional nanostructures and nanomaterials for effective harnessing and storage of clean energy from sunlight, purifying water and air for clean environment and imaging and spectroscopy and nanofabrication using ultrafast laser towards fast-speed all-optical communications and intelligent manufacturing. She is an elected Fellow of Optica (formally known as OSA) and an elected Fellow of the Institute of Materials, Minerals, and Mining. She serves on the College of Expert for the Australian Research Council since 2019. Now please follow Light scientific editor to enter Prof. Jia’s academic world.

**1. First of all, we really appreciate your great support to** ***Light*** **and** ***LAM***. **You are a leading expert in the field of integrated and nanophotonics and has taken the lead in organizing the** ***LAM*** **special issue on integrated photonics. What are your plans and expectations for this special issue?**

Integrated photonics is a fast expanding field. Fueled by the tremendous advancement in nanofabrication, in the past decade, there have been amazing breakthroughs on both fundamental sciences and impactful technologies in integrated photonics. And nowadays, integrated photonics is playing more and more critical roles in communications, biomedicine, sensing, computing, automotive and metrology and so on. Through this special issue, we are hoping to capture these exciting advancements. And in the meantime, to gather the leading experts in the field to prospect some key insights of the remaining challenges and to point out where the field is heading for. By doing so, we hope more synergistic effort can be put into this critical area and facilitate some major breakthroughs in the advanced manufacturing field.

**2. You have published a paper “Diffraction-limited imaging with monolayer 2D material-based ultrathin flat lenses” in Light** ^1^ **, which was the cover paper of issue 5 in 2020. The cover image is very beautiful. Can you introduce the major breakthroughs made in this research achievement and the inspiration of designing cover image at that time?**

First of all, I would like to sincerely thank *Light* for providing us such a wonderful platform for disseminating our research findings. This particular work was in collaboration with Prof. Qiaoliang Bao, Prof. Chengwei Qiu, and Prof. Kian Ping Loh on laser nanoprinting of a high performance flat lens on the thinnest possible material, which was a monolayer of transition metal dichalcogenides (TMDC) with a thickness of a few angstroms. The work was really driven by the urgent need of integrated photonics chip, on which each component needs to be miniaturized to achieve the fastest speed, maximum bandwidth and minimum power consumption. The ultimate miniaturization will be on a single atomic layer. By demonstrating the imaging function on this monolayer of TMDC, the feasibility of manipulating material at atomic accuracy by laser nanoprinting has been proved, opening new opportunities for laser advanced manufacturing at unprecedented accuracy.

The monolayer lens we fabricated not only can achieve three-dimensional diffraction-limited focal spot, but also can be used in the real life imaging. In our paper, we demonstrated the imaging of standard target to demonstrate the high resolution. With the capability of this flat lens, many ambitious applications can be envisaged. Butterfly is a symbol of beauty. On the front cover the lens is used to image a colorful butterfly, which indicates this monolayer flat lens can help to reveal the beautiful microworld within a compact configuration.

**Figure 1:**
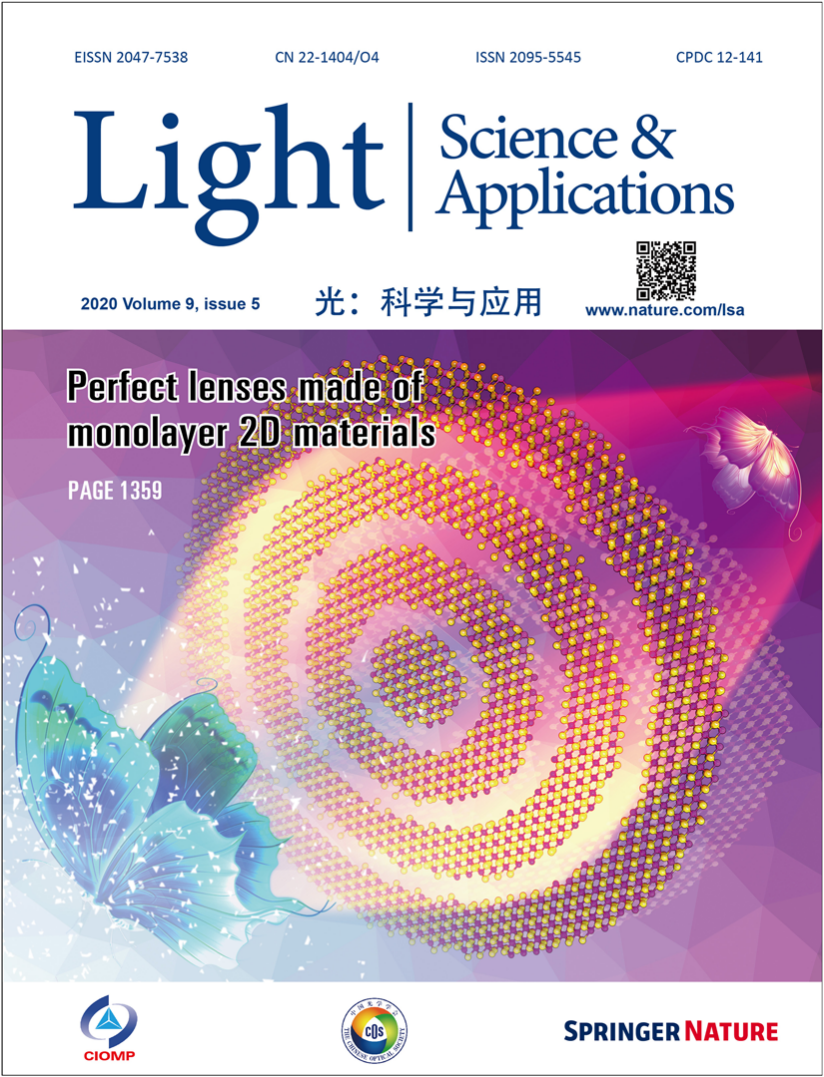
The cover of Light’s 2020 Volume 9, Issue 5

**3. In recent years, graphene and other two-dimensional materials have attracted much focused research. You are a leading expert in this field and have published a series of relevant papers. What is your view on the future of this field, and where do you think is the next breakthrough of meta-materials?**

Indeed, graphene and a large number of other two-dimensional materials, which we call atomaterials, have emerged as wonderful candidates for optoelectronic devices in the past decade. They have outstanding physical or chemical properties not available in their bulk counterparts. These materials have created a lot of exciting new opportunities to solve the challenging problems that we could not solve before. Together with my peer researchers worldwide, my team would like to contribute to the development of this emerging field. We have established a research center at RMIT University dedicated to atomaterial sciences and technologies research. Tremendous researches have shown that the hybridization of two-dimensional materials with existing device architecture can significantly boost their performance. We believe further developing nanofabration techniques on two-dimensional materials can effectively engineer materials on the atomic level and lead to metadevices with more desired and designed properties. We have a recent paper called “Engineering van der Waals Materials for Advanced Metaphotonics” published on Chemical Review with Prof. Chengwei Qiu, which elaborates our thoughts on this aspect^2^.

In the meantime, atomaterial is an emerging but fast expanding field. It requires the effort from many different disciplines, including chemistry, physics, material and engineering. To facilitate the free communications between different fields, I have been pushing very hard the concept of “Open Lab”, which not only includes academia but also industry and government. Only when there is clear driving force from the industry, there is government policy and fund to facilitate, the scientific breakthroughs from labs can produce some real productivity and impact everyone’s life.

**Figure 2:**
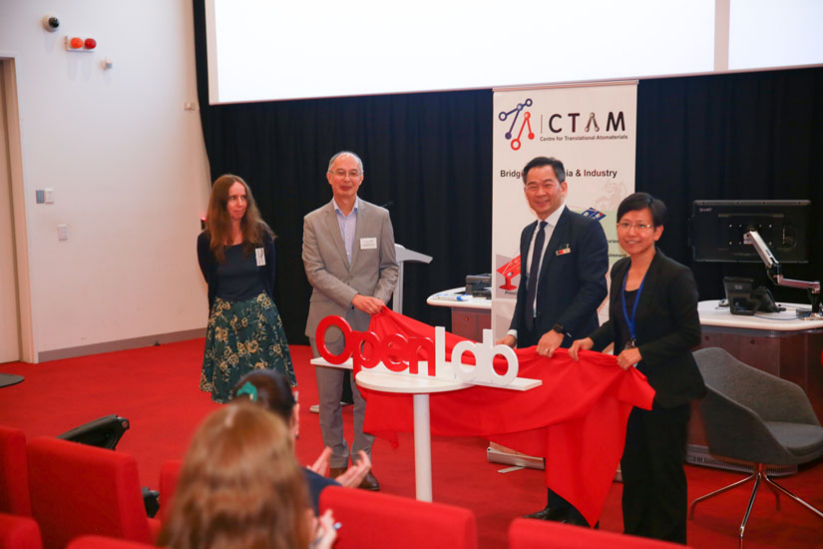
Open Lab opening ceremony featuring Prof. Baohua Jia (right), Prof. Alan Lau (Pro Vice-Chancellor (Research Partnership and Digital Innovation, Swinburne University of Technology)), Dr. Rober Mun (Executive Director, Australian Research Council), and Prof. Beth Webster (Pro Vice-Chancellor (Research Impact and Translation, Swinburne University of Technology, left))

**4. You are the judge of this year’s (2022) Light Doctoral Academic League of Optics and Optical Engineering. How did the contestants perform this year? Did they leave a deep impression on you?**

It was an absolute honor to have the opportunity to participate in the 2021 and 2022 Optics and Optical Engineering Doctoral Academic Competition hosted by *Light*, and to contribute to the competition as a judge.

My deepest feeling about the contestants is that their research works are truly outstanding, and many achievements have reached world-leading level. Most of the reported work is not only cleverly conceived, innovative in basic research, but more importantly, has breakthroughs in practical applications. A number of them have realized prototypes with good performance and solving problems in actual production and real life. This is exactly the purpose of scientific research. I would like again to congratulate the young researchers on their achievements, and I was very glad for the challenges they brought to our jury panel.

Nowadays, most people are deeply aware of the importance of science and technology, especially high precision optical technology in production and life. As optical scientists, we are caught up in the best of times. I hope that the students can concentrate on research, make persistent efforts, challenge themselves to climb the peak of scientific research, and be determined to lead the world. In the meantime, I would like to thank *Light* for providing such a fair and impartial competition platform. I sincerely hope that the light competition will become the cradle of cultivating and identifying top optical talents.

**5. You are the chairman of the Australia-China Advanced Materials and Manufacturing Association, elected Fellow of Optical Society of America (OSA) and elected Fellow of the Institute of Materials, Minerals, and Mining (IMO3). Why are you so keen to participate in such activities? How do you evaluate the development of China’s advanced manufacturing?**

In my spare time, I am very passionate to participate in professional activities and contribute my energy to the society. I believe scientists are people who break boundaries and create new possibilities and opportunities. However, this can’t be done by a single person and has to rely on team effort. Through connecting with different people from different backgrounds, innovative ideas can be generated and problems can be solved. Therefore, I believe it is my mission to help to create these connection networks and platforms so that more good ideas can be generated and more problems can be solved.

The development of China’s advanced manufacturing meets the urgent needs of the society. But it requires tremendous effort from various sectors to make it happen. China has a huge market, which provides a great driving force for technological development. Technology advancement requires many years of accumulations on fundamental scientific breakthroughs and consistent effort on engineering. I think the prosperity of optical science and engineering is a good sign that alike disciplines are now getting noticed and chosen by young people. The more talented young people are in these areas, the faster advanced manufacturing is progressing.

**6. COVID-19 has changed our lives greatly. Many scientific research projects have been interrupted or delayed. Online international conferences have become the new norm. Personally, do you prefer online conferences or offline? Could you give some suggestions or advice to help researchers go through this unusually tough period?**

This really depends on the situation. I guess for conferences, most people prefer face to face interactions because it creates the personnel links between people and allows in-depth communications. But it is indeed challenging during COVID period. To be honest, at the beginning of COVID, I wasn’t so used to online meetings, but later on I found that although online meetings could not build up too much personnel relation, it is an effective way of disseminating messages. For example, like the Light competition, if it is offline, its impact will be only limited to those who attended the event. But through online, there were several tens of thousands of people watching the event. So, I guess, if one wants to create personnel links with other people, try to meet in person. But for quick and effective disseminating of messages, online meeting can be a good approach.

**7. You received your Ph.D. degree from Swinburne University of Technology. What do you think you have gained from your PhD studies? What impact did it have on your subsequent research career?**

I think the best part I gained from my PhD is the critical thinking and problem-solving capability. During PhD, one needs to solve problems which have not been solved before. Although there is methodology to follow, there is no existing solution for an easy answer. The solution needs to be found. During this process, many skills have been developed, including analyze the problem, develop a methodology, develop a plan, communicate with relevant people, develop theory and experiment, resource management, data analysis, paper drafting, and dissemination and rebuttal et al. During this process, one needs to think through the problem and develop solution. So it was an intensive training process. If you go through it and become a PhD, that means you are ready to face any complex problem, no matter it is research related or not, and have a mindset and methodology to solve it. The PhD study has benefited my whole research career.

**8. What obstacles or difficulties have you encountered in your research work? How did you overcome these difficulties?**

The research life is actually quite enjoyable, although there is always challenging scientific problems to solve. But that is the beautiful part of research, solving challenges all the time. For me, the difficulties are not the research itself, it is actually the communications. I found as a scientist, I often talk too much about technical terms, which lay person find hard to understand. This is a big problem because letting people know what we are doing and how we can change the world is very important because they actually pay their tax to support our research.

To solve this problem, I have tried to talk to different people whenever I can, a taxi driver, a cleaner, a security or an administrative colleague. I also deliver public talks and visit high schools regularly. To help me communicate better to ordinary people, I also attended media training classes and learn from experienced speakers. These trainings help a lot.

**9. In your life and career, who has the biggest influence on you, and in what way?**

My high school teacher changed my attitude towards physics. When I was at year 10 of high school, I thought physics was very difficult, and it wasn’t an easy subject for girls, which was the common view. At the beginning of year 10, only one person passed the first physics exam we had. But our teacher, Mr. Liu, who actually designed that exam, changed my mind. He told us, “You all think physics is very difficult. That’s why you didn’t pass it!” Eventually, he taught us the right way to approach this subject. He pointed out that it wasn’t necessary to remember all the equations because memorizing doesn’t mean you really understand things. He thought it was more about the attitude and approach to solving problems, so that was what he taught us to do. He said, “As long as you’re confident, and you have the passion to work on it and solve it, you’ll be all right”. After a couple of months, he gave us that same exam again, and most of us passed! He changed my mind entirely, and I became very interested in physics. I like to solve problems, fix things and get things done, so it actually suits my personality very well. I also realized that ability in physics isn’t necessarily related to gender; it’s more about wanting to study it. So when I finished my entrance exam to do undergraduate study at my college, I decided to study physics, because I wanted to be a good physics teacher like Mr. Liu. I think it’s very important to inspire people.

My PhD supervisor, Professor Min Gu, is a wonderful and influential role model for me. I’m always inspired by his vision and ideas. He’s very helpful and has lots of strategies for managing research and managing teams—the important aspects of being a good researcher. I think the most important thing I’ve learned from him is dedication—if you believe in something, you don’t question it but just dedicate yourself to it, no matter how hard it sometimes is. I’ve understood that better in recent years. The difference between a successful person and a loser is determination, persistence, and dedication.

**10. How do you balance your work and family life?**

I am very lucky to have a husband who also works as a researcher (although in a different field). Because he understands my work requirements, he supports me. I need to travel a lot to meet industry partners and attend conferences and workshops, and I work very long hours sometimes. He has been very supportive. I love motherhood, it makes my life complete. But getting the right balance can be challenging. I feel that if you’re really passionate about your career, you’ll find some way to balance it. Actually, it’s the same for everything. It’s always hard, but as long as you’re positive, and you feel it’s worthwhile, there will be a solution. For me, I have been trying to talk about my work with my kids, allowing them to understand what I have been doing. I also visit my kids’ school to make science outreach, which makes my kids very proud. On university open days, I also invite my family to visit my lab and show them my projects. These activities help them to get familiar with what I do and make them feel they are part of my career. I also have to say, I have a great team to support me in my career. They all do a great job, which relieves me from heavy work.

**Figure 3:**
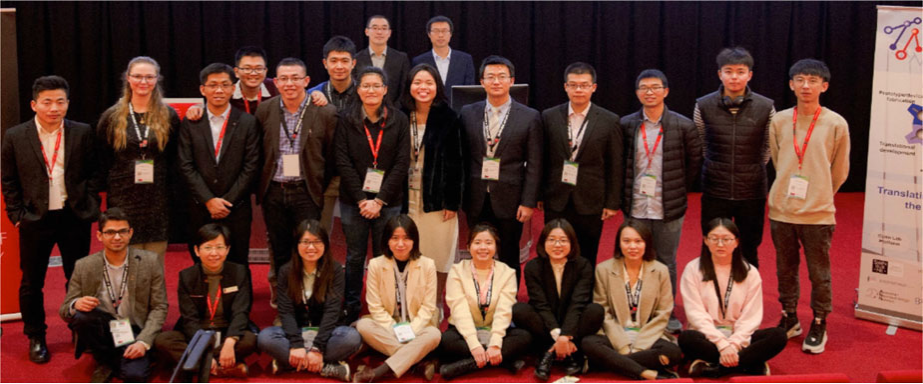
Prof. Baohua Jia’s team

**11. What advice do you have for young researchers?**

I think the first—and probably most important—message is a positive attitude. Having a positive attitude will enable them to achieve many things and don’t waste energy distracting themselves from finding solutions. At graduation gatherings, my students have actually told me that I’ve changed their attitude to one of positivity. I’m always glad to hear that!

The second message is passion. One needs to be passionate about what he/she is doing. If he/she is just doing it because the feeling of having no choice, that’s never going to work. So when I interview potential PhD students, I always ask, “Why do you want to do a PhD?” If they tell me that they’re really fascinated about a particular question, or they have an idea they want to turn into something that nobody in the world has yet done, and that’s what inspires them, I will be happy to take them, otherwise, I will ask them to seriously think about the serious commitment.

And the third message is commitment. It’s very important for a successful career. In our field, there’s always a period of struggle for early career researchers, because sometimes they can’t see where the lighthouse is. They find it difficult to get funding, to get a fellowship, or to publish papers, and the competition is so fierce. So they are tempted to go out and find a better-paid job! But if you’re really passionate about it, you commit and just keep going, you will succeed.

The fourth thing I like to emphasize to young researchers is the importance of getting engaged with society and with other researchers, both locally and internationally. Of course, you need to focus on your research and do it very well, but outreach and networking are very important. When you actually apply for a grant, of course they’ll look at your track record, but they’ll also look into whether this person is really someone who can produce an impact that is recognized by other people. This requires a lot of, I won’t call it self-promotion, but lots of communication. You need to tell people what you’re doing, what you’re fascinated about.

And last but not least, you need to have the confidence to apply for awards. I’ve received quite a few, but it’s not really because I’ve done something better than others; it’s because I applied! If you don’t apply, you won’t get it—that’s for sure! So be brave. Often there are many people qualified to get that award, but they might not think they’re competent enough, so they don’t even try. And if you get rejected, try to find out why that was happening so that you’ve learned something for next time.

**Figure 4:**
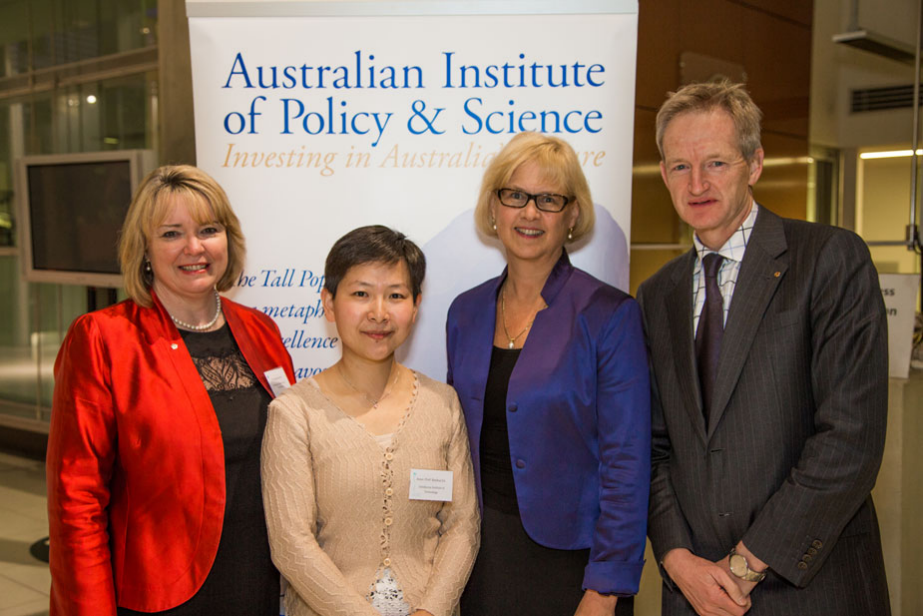
Prof. Baohua Jia received the Young Tall Poppy Award from Australian Institute of Policy & Science

**Short Bio**

*Professor Baohua Jia is an Australian Research Council Future Fellow and Director of Center for Atomaterials Sciences and Technology in School of Science at RMIT University. Before joining RMIT, Prof. Jia was the Founding Director of Center for Translational Atomaterials at Swinburne University of Technology. Her research focuses on the fundamental light and nano-and atomaterial interactions. In particular, her work on laser manipulation of two-dimensional materials has led to the design and fabrication of functional nanostructures and nanomaterials for effective harnessing and storage of clean energy from sunlight, purifying water and air for clean environment and imaging, spectroscopy and nanofabrication using ultrafast laser towards fast-speed all-optical communications and intelligent manufacturing. Prof. Jia has co-authored more than 260 scientific publications in highly ranked journals and prestigious international conferences. She has delivered more than 60 keynote/invited talks at international conferences and serves multiples professional committees and boards. She is an elected Fellow of Optica (formally known as OSA) and an elected Fellow of Institute of Materials, Minerals and Mining. Since 2019, Prof. Jia has been serving as the Australian Research Council College of Expert. She has received numerous prizes and awards, including the ARC Future Fellow, DECRA and APD, Finalist for the Prime Minister’s Science Awards, Young Tall Poppy Science Award, and L’Oréal Australia and New Zealand for Women in Science Fellowship et al.*

**Figure:**